# Explainable artificial intelligence for prediction of refractory ulcerative colitis: analysis of a Japanese Nationwide Registry

**DOI:** 10.1080/07853890.2025.2499960

**Published:** 2025-05-05

**Authors:** Masaya Sano, Yasuhiro Kanatani, Takashi Ueda, Shota Nemoto, Yurin Miyake, Naoko Tomita, Hidekazu Suzuki

**Affiliations:** a Department of Gastroenterology, Tokai University School of Medicine, Isehara, Kanagawa, Japan; b Department of Clinical Pharmacology, Tokai University School of Medicine, Isehara, Kanagawa, Japan; c Industrial & Digital Business Unit, Hitachi Ltd, Chiyoda-ku, Tokyo, Japan

**Keywords:** Ulcerative colitis, artificial intelligence, nationwide registry

## Abstract

**Objective:**

Ulcerative colitis (UC) is a chronic inflammatory bowel disease for which remission is dependent on corticosteroid (CS) treatment. The diversity of disease pathophysiology necessitates optimal case-specific treatment selection. This study aimed to identify prognostic factors for refractory UC using a machine learning model based on nationwide registry data.

**Methods:**

The study included 4003 patients with UC with a Mayo score of ≥3 at the time of registration who had been using CS since their entry out of 79,096 newly registered UC cases in a nationwide registry from April 2003 to March 2012 (before the widespread use of biologic agents in Japan) with 3-year data. A pointwise linear (PWL) model was used for machine learning.

**Results:**

A PWL model, which was developed to predict long-term remission (lasting >3 years), had an area-under-the-curve (AUC), precision rate, recall rate, and F-value of 0.774, 0.55, 0.70, 0.62, respectively, in the test dataset from the time of registration to 2 years later. Furthermore, the presence of pseudopolyps at the time of registration was significantly and negatively correlated with remission, highlighting its importance as a prognostic factor.

**Conclusions:**

In this study, we constructed a highly accurate prognosis prediction model for UC, in which inflammation persists for an extensive period, by training a machine learning model for long-term disease progression. The results showed that machine learning can be used to determine the factors affecting remission during the treatment of refractory UC.

## KEY MESSAGES

A machine learning model was developed for patients who underwent CS therapy during remission induction, with remission as the outcome in the third year after treatment.

The precision of the machine learning model is evident in its ability to categorize the cases into two groups with a different predicted probability of remission.

A comparison of predicted and actual remission showed that the absence of pseudo-polyps and early CS withdrawal influenced remission.

## 1. Introduction

Ulcerative colitis (UC) is an intractable inflammatory bowel disease characterized by severe diarrhea, bloody stools, severe abdominal pain, and fever with relapse and remission. It is caused by genetic, immunological, and environmental endogenous modifying factors [1]. The main features are cellular infiltration of the colonic mucosa and structural disruption of the crypts [2]. UC diagnosis is confirmed by clinical, endoscopic, and histological examinations, and it presents with various pathologic manifestations [3]. Based on these findings, the clinical stage of UC is determined and treatment is selected according to the background of the case [4].

According to the Japanese treatment guidelines, corticosteroids (CS) induce remission in patients with mild-to-moderate UC who do not respond to 5-ASA. However, if the patient shows resistance to CS, the treatment shifts to moderate-to-severe UC management. In moderate-to-severe UC, remission is typically induced by CS, followed by cytapheresis (CAP), immunosuppressants, biological agents, and small-molecule kinase inhibitors [5].

While CS are effective for inducing remission, they are unsuitable for maintaining it [6]. Long-term use of CS should be avoided due to the risk of adverse events and complications, including impaired glucose tolerance and osteoporosis [7].

Therefore, switching to medications that do not depend on CS is essential. Additionally, the effectiveness of CS must be evaluated during the remission induction phase. Factors such as a history of smoking, low hemoglobin levels, and elevated calprotectin levels in the stool are recognized as risk factors for failure of CS therapy [8,9]. When using CS to induce remission, predicting the response of UC based on the patient’s condition allows switching from CS to biological agents [10]. A large-scale claims database study in Japan revealed that long-term CS use was most closely associated with low initial CS dose [11]. Factors related to CS overuse or dependence include moderate-to-severe disease activity in UC, and thiopurine monotherapy [12].

Various studies have examined treatment options with which disease types achieve higher remission rates; however, the diversity of UC has limited the development of all-inclusive predictive models [13]. A machine learning model has been proposed that, after using medical records, can select cases with characteristics closest to a particular case and identify the optimal treatment for remission [14]. However, conventional methods for constructing such a model are limited in their ability to perform analysis while maintaining the relationship between explanatory and objective variables, making it difficult to assign meaning to the obtained results. In this regard, we showed that performing an analysis while preserving the relationship between the explanatory and target variables is possible using a pointwise linear (PWL) model for machine learning [15].

Japan has a public subsidy system for patients with UC. Approval for the subsidy is obtained by submitting the prescribed clinical information [16]. Therefore, nationwide clinical information on patients with UC who have submitted subsidies has been accumulated. Using large-scale data on patients with UC from a period when biological agents were not common, we aimed to construct a remission prediction model for cases where CS was required from the time of remission induction and to clarify the factors involved in remission.

## 2. Materials and methods

### 2.1. Data source and collection

Data were obtained from application forms (Supplementary Table S1) submitted to and digitized by the Ministry of Health, Labor, and Welfare (MHLW) between April 2003 and March 2015. Duplicate cases and those without essential demographic data such as age of onset were excluded. The patients included in the analysis fulfilled the diagnostic criteria for UC established by the study group for intractable inflammatory bowel disorder, which aligns those of the MHLW.

### 2.2. Study participants

**Figure 1.**
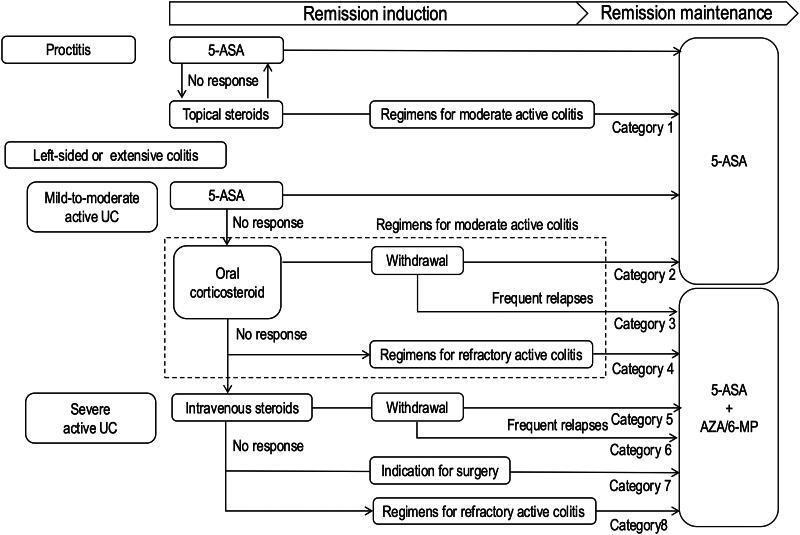
Treatment algorithm for UC. Regimens for refractory active colitis included CPA, tacrolimus, cyclosporine, and biologics. AZA: azathioprine; 6-MP: 6-mercaptopurine.

Patients diagnosed with proctitis, left-sided colitis, or extensive colitis ranging from mild to severe at the time of registration, and who required CS medication for the induction of remission were included in this study. In this context, refractory UC is defined as active UC that did not achieve remission with 5-ASA and maintained a Mayo score of ≥3 3 years after being transitioned to CS-based therapy. The clinical progression of these cases was classified into categories 1–8, as illustrated in Figure 1.

### 2.3. Disease distribution and clinical assessment

Clinical assessment was performed using the Mayo score, and clinical remission was defined as a Mayo score <3 [17]. Disease distribution was determined using the Montreal classification as follows: E1, ulcerative proctitis; E2, left-sided UC; and E3, extensive colitis [18]. A case not in the Montreal classification system was defined as ‘Other’.

### 2.4. Machine learning model

A PWL model (implemented in Pytorch 1.5.1 [Linux Foundation], Python 3.7.4 [Python Software Foundation]) was used to predict the induction of remission after 3 years and patient stratification [15,19]. The PWL model is an explainable machine learning method that provides a weight vector tailored to each sample. The weights in a PWL model are calculated as nonlinear functions of the features through its machine neural network, which differs from logistic regression. The feature variables of the dataset were classified as binary (B), category (C) or quantitative (Q) (Supplementary Table S2). Binary variables were encoded as 1 or −1. Quantitative variables were normalized (mean = 0; ­standard deviation = 1). After this encoding and normalization process, the missing values were replaced with zero because a zero input value does not change the output in the weighted-sum layers of the machine learning model. Therefore, the zero-value input does not change the inference of the model. Furthermore, 10-fold double cross-validation (DCV) was performed to optimize the hyperparameters and evaluate the predictive performance of the model. The model with the best prediction accuracy (the highest area under the curve (AUC) of the test set in the 10-fold DCV) was used for patient stratification. The optimal hyperparameters are listed in Supplementary Table S3.

### 2.5. K-means clustering algorithm for UC

Given the diversity of UC, the patients in this study may have been divided into subgroups depending on their features. Therefore, we performed clustering using a weight vector tailored for each patient in the PWL model. The weights were stratified into *K* = 2 clusters using the K-means + algorithm (implemented in Scikit-learn 0.24.2) [20]. The elbow method was used to select K (i.e. the appropriate number of clusters) [21].

### 2.6. Statistical analysis

Descriptive statistics are reported as counts (percentages) and have been used to describe the characteristics of the cases included in the analysis. Kruskal–Wallis one-way analysis of variance by rank test was performed to compare cluster variables. All *p*-values are reported to three decimal places, with *p* < 0.001 reported as *p* < 0.001. χ^2^ tests were used to compare categorical variables. Residual analysis was performed to determine which cell numbers in the cross-table represented sources of bias (*p* < 0.05) when significant bias was observed in a χ^2^ test (*p* < 0.05). All analyses were performed using STATA ver. 18.0 (Stata Corporation LLC, College Station, TX, USA).

## 3. Results

### 3.1. Demographic data

**Figure 2.**
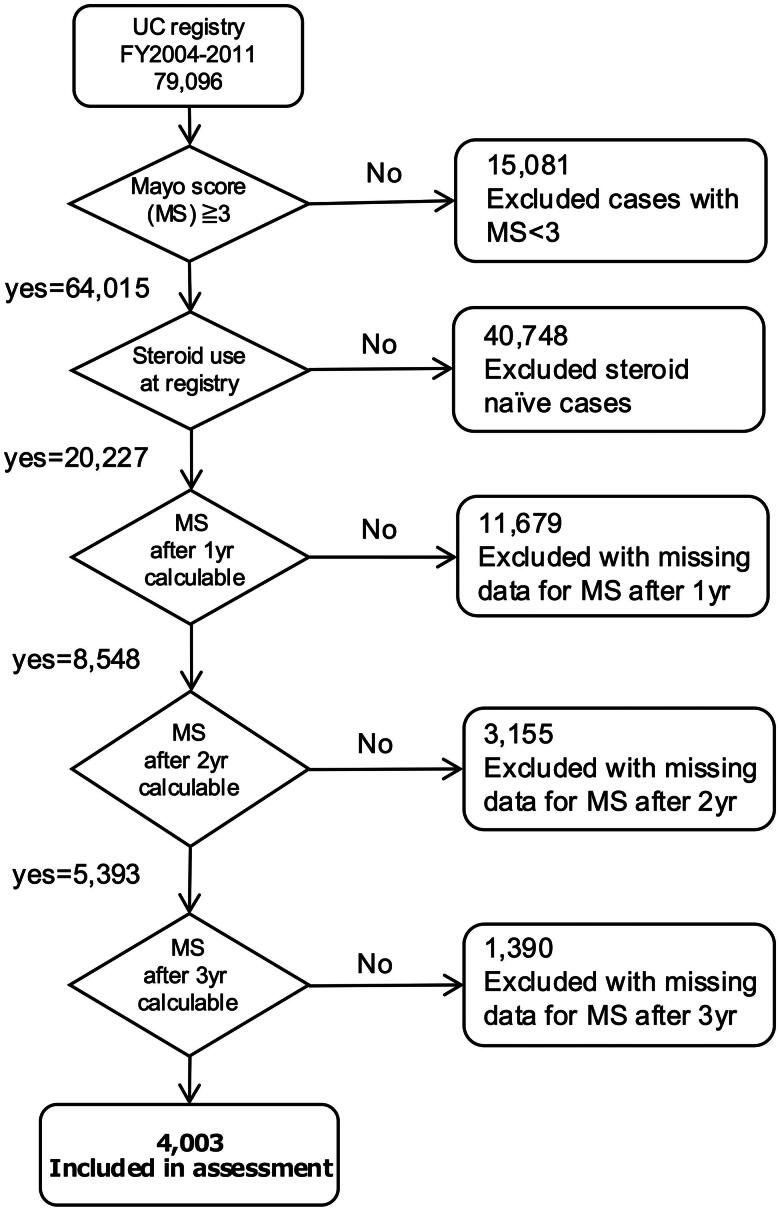
Flow diagram of study participants.

Of the 79,096 patients identified, 4003 with 3 years of data, a Mayo score ≥3 at the first visit, and use of CS from the first visit were included in the analysis (Figure 2). Of the 4003 cases, 2630 did not achieve remission at 3 years and were classified as refractory UC, while 1373 cases reached remission within the same timeframe.

Table 1 compares the two groups in terms of the rate of remission achieved by the third year after registration. The non-remission group exhibited a significantly higher age at registration; however, no significant differences were noted in terms of age at onset or gender. Laboratory findings revealed that the non-remission group had elevated anemia, and levels of white blood cell (WBC) count, C-reactive protein (CRP), and erythrocyte sedimentation rate (ESR), along with significant decreases in total protein (TP) and albumin (Alb). The clinical findings indicated that individuals in the non-remission group experienced more frequent bowel movements and greater abdominal pain. In terms of the extent of disease, no significant differences were found between the two groups. Whereas, the endoscopic findings showed a considerably higher prevalence of pseudopolyps in the non-remission group than in the remission group (18.1% vs. 8.8%, *p* < 0.001). In contrast, no significant differences were observed in biopsy findings, such as cell infiltration, erosion, and cryptal abscess. The Mayo score revealed a notable trend towards a higher incidence of moderate severity in the non-remission group (46.5% vs. 56.8%, *p* < 0.001). Details of the treatment progress of the patients (Figure 1) are shown in Table 2. The remission rate after 3 years was the highest in case 2 (41.0%) and lowest in case 3 (25.1%).

**Table 1.** Characteristics of ulcerative colitis (UC) cases requiring corticosteroids (CS) since registry entry.

	Remission at 3 years		
Variables	Yes (*n* = 1373)	No (*n* = 2630)	*p*-value
Age at registration (years)	41 ± 16	42 ± 17	0.01
Age at onset (years)	39 ± 16	40 ± 18	0.09
Female (%)	42.0	42.1	0.99
Laboratory findings			
RBC (×10^4^/μL)	444 ± 58	430 ± 64	<0.001
Hb (g/dL)	13.0 ± 2.2	12.5 ± 2.3	<0.001
WBC (/µL)	7,966 ± 3,590	8,340 ± 3,637	0.002
CRP (mg/dL)	3.1 ± 6.4	3.7 ± 6.3	0.004
ESR (mm/h)	29 ± 31	32 ± 33	0.006
TP (g/dL)	6.9 ± 0.7	6.8 ± 0.8	<0.001
Alb (g/dL)	3.9 ± 0.7	3.7 ± 0.8	<0.001
Stool culture (%)	7.5	8.6	0.28
Clinical findings			
Body temperature (°C)	37 ± 0.8	37 ± 0.9	0.001
Pulse (/min)	80 ± 15	82 ± 15	0.001
Defecation (times/d)	6.3 ± 7.3	7.1 ± 5.2	<0.001
Abdominal pain (%)	48.5	56.7	<0.001
Extent of lesions			
E1	373 (27.2%)	591 (22.5%)	0.506
E2	1252 (30.9%)	424 (31.5%)	
E3	536 (39.0%)	1114 (42.4%)	
Other	40 (2.9%)	97 (3.7%)	
Endoscopic findings			
Loss of vascular markings (%)	97.6	97.3	0.564
Easy bleeding (%)	93.9	93.8	0.863
Mucosal fragility (%)	95.1	95.7	0.412
Erosion (%)	90.8	92	0.221
Pseudopolyps (%)	8.8	18.1	<0.001
Contiguous lesion (%)	82.6	85.5	0.02
Biopsy findings			
Cell infiltration (%)	98.1	98.7	0.16
Erosion (%)	88.3	90.2	0.067
Crypt abscess (%)	73.6	71.7	0.223
Reduced no. of goblet cells (%)	69.7	71.9	0.156
Mucosal abnormality (%)	38.1	40.5	0.163
Dysplasia (%)	7.5	8.1	0.555
Mayo score			
Mild: 3–5 (%)	53.2	42.8	<0.001
Moderate: 6–10 (%)	46.5	56.8	
Severe: 11–12 (%)	0.2	0.3	
			

*n*: number; RBCs: red blood cells; Hb: hemoglobin; WBC: white blood cells; CRP: C-reactive protein; ESR: erythrocyte sedimentation rate; TP: total protein; Alb: albumin.

**Table 2.** Clinical progression of UC cases.

Clinical progression		0_year					1_year					2_year					Remission_3y (%)
	No. case	CS	5-ASA	IS	CAP	Surgery	CS	5-ASA	IS	CAP	Surgery	CS	5-ASA	IS	CAP	Surgery	
Category 1	964	964 (100)	856 (88.8)	16 (1.7)	16 (1.7)	8 (0.8)	484 (50.2)	650 (67.4)	20 (2.1)	4 (0.4)	2 (0.2)	377 (39.1)	703 (72.9)	76 (7.9)	20 (2.1)	7 (0.7)	373 (38.7)
Category 2	644	644 (100)	592 (91.9)	32 (5.0)	21 (3.3)	4 (0.6)	300 (46.6)	592 (91.9)	34 (5.3)	8 (1.2)	10 (1.6)	0	567 (88.0)	82 (12.7)	13 (2.0)	6 (0.9)	264 (41.0)
Category 3	1119	1119 (100)	1038 (92.8)	35 (3.1)	34 (3.0)	5 (0.4)	717 (64.1)	732 (65.4)	53 (4.7)	4 (0.4)	11 (1.0)	741 (66.2)	758 (67.7)	189 (16.9)	43 (3.8)	5 (0.4)	281 (25.1)
Category 4	1265	1265 (100)	1160 (91.7)	46 (3.6)	44 (3.5)	6 (0.5)	884 (54.9)	884 (69.9)	64 (5.1)	6 (0.5)	7 (0.6)	558 (44.1)	943 (74.5)	170 (13.4)	30 (2.4)	15 (1.2)	452 (35.7)
Category 5	3	3 (100)	3 (100)	2 (66.7)	0	0	0	2 (66.7)	0	0	0	0	2 (66.7)	1 (33.3)	0	0	1 (33.3)
Category 6	4	4 (100)	4 (100)	1 (25.0)	0	0	3 (75)	4 (100)	0	0	0	4 (100)	4 (100)	2 (50.0)	0	0	2 (50)
Category 7	3	3 (100)	3 (100)	0	0	0	3 (100)	2 (66.7)	1 (33.3)	1 (33.3)	3 (100)	1 (33.3)	0	0	0	0	0
Category 8	1	1 (100)	1 (100)	0	1 (100)	0	1 (100)	0	0	0	0	0	0	0	0	0	0

CS: corticosteroids; 5-ASA: 5-aminosalicylic acid; IS: immunosuppressants; CAP: cytapheresis.

### 3.2. Construction of a prognosis prediction model and verification of prediction accuracy

**Figure 3.**
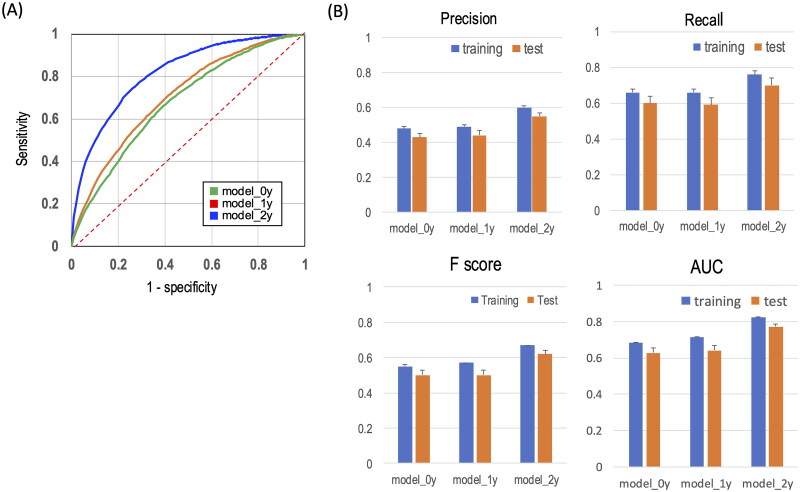
Evaluation of a machine learning model for the prediction of remission. (A) AUC for each prediction model. The dashed red line represents the random classifier, with an AUC value of 0.5. (B) Precision, recall, and *F-score* for each prediction model. The blue and orange columns are for the training and test data sets, respectively.

We evaluated three prognostic models: one based on information collected at the time of registration (model_0y), one using data up to 1 year after registration (model_1y), and one using data up to 2 years after registration (model_2y). We used the AUC and F as performance metric scores to assess the model accuracy. The AUC values in the test dataset of the mean 10-fold DCV for each model were 0.628, 0.641, and 0.774 for model_0y, model_1y, and model_2y, respectively (Figure 3(A)). For model_2y, the precision, recall, and F-score using the test dataset were 0.55, 0.70, and 0.62, respectively (Figure 3(B)).

### 3.3. Prognostic factors related to remission

**Figure 4.**
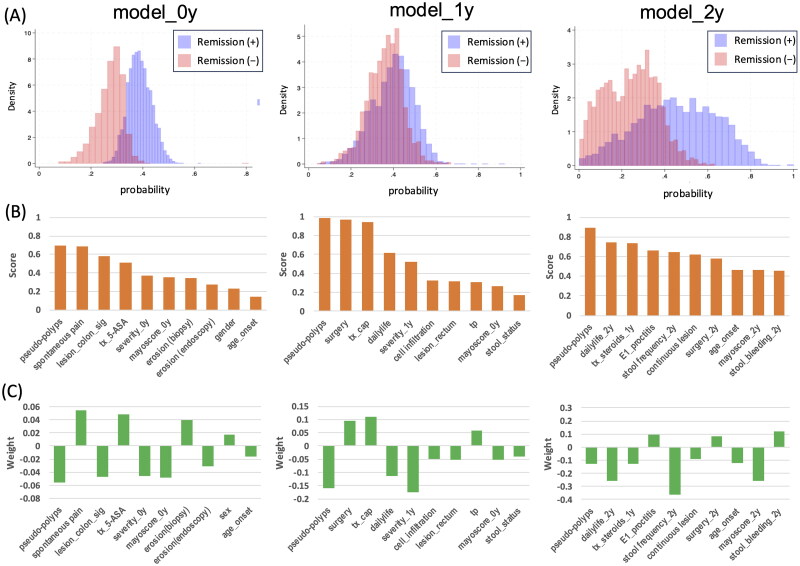
Classification with remission as the outcome and analysis of prognostic factors. (A) Histogram of remission prediction rate. Blue and red bars indicate the remission and non-remission prediction groups, respectively. (B) Correlation coefficients (score) of the top 10 features based on factor analysis. (C) Contribution of important factors to outcome (weight).

Figure 4 shows the clustering results for each model. As we progressed from model_0y to model_1y and then to model_2y, the distinction between groups with high predicted remission rates (highlighted in blue) and those with low predicted remission rates (highlighted in red) became increasingly evident. When examining the significant factors related to remission using only data from the registration year, several factors were found to be highly correlated, including pseudopolyps (0.695), abdominal pain (0.689), sigmoid colon inflammation (0.578), and the use of 5-ASA (0.513). The weight values for these factors were negative for both pseudo-polyps (−0.056) and abdominal pain (−0.054), indicating a potentially negative effect on remission rates. Conversely, sigmoid colon inflammation (0.054) and the use of 5-ASA (0.049) showed positive weight values, suggesting a potentially beneficial effect. When analyzing data from the registration year to the following year, the key factors identified were pseudo-polyps (0.982), surgery (0.964), and CAP (0.940). The weight values revealed a negative correlation for pseudopolyps (−0.159) and positive correlations for surgery (0.094) and CAP (0.109), further emphasizing their potential influence on remission rates. For data extending up to 2 years after registration, significant factors included pseudo-polyps (0.893), daily life in the second year (0.743), and the use of CS in the first year (0.736).

### 3.4. Effect of treatment choice on remission prediction

Predictive modeling through machine learning revealed that treatment-related factors influencing remission at three years may include 5-ASA (year of registration), type of surgical operation (year |of ­registration), CAP (year of registration), and CS administration (1 year later). Subsequently, a logistic regression analysis was performed on the outcomes related to achieving remission at 3 years, utilizing 5-ASA, surgery, CAP, and CS administration as key factors in the analysis.

The odds ratios for 5-ASA, surgery, CAP, and CS administration were 1.56 (95% confidential interval (CI), 1.14–2.13, *p* = 0.005), 0.12 (95% CI, 0.02–0.90, *p* = 0.039), 0.81 (95% CI, 0.49–1.34, *p* = 0.410), and 0.56 (95% CI, 0.48–0.67, *p* < 0.001), respectively.

## 4. Discussion

Notably, 5-ASA is the preferred initial treatment for mild to moderate cases of proctitis, left-sided colitis, and total colitis in UC, with CS administered if remission is not achieved [22]. Two-thirds of patients treated with CS require additional CS treatment, and to establish the efficacy and safety of CS for the induction of remission in moderately to severely active UC, the European Crohn’s and Colitis Organization guidelines recommend limiting CS use to a maximum of 3 months and using CS-sparing agents for relapses associated with CS tapering [23]. These guidelines state, ‘there is less evidence regarding the importance of early treatment initiation in UC than Crohn’s disease. At the same time, repeated flare-ups of symptoms can lead to physical and psychological disability, as can repeated exposure to CS. On the other hand, it is clear that appropriate and timely selection of patients for high-cost interventions is important to achieve optimal health economic outcomes; the ultimate goal of treatment in UC is to maintain health-related quality of life and avoid disability’ [24,25]. In diseases such as UC, where pathological transitions vary significantly from case to case, using standard treatments based on the symptoms, extent, and severity of lesions at the initial diagnosis is complex [26].

When comparing the two groups regarding their achievement of remission after 3 years, the non-remission group exhibited significantly higher levels of intestinal inflammation than the remission group. Notably, the occurrence of pseudopolyps was more prevalent in the non-remission group; however, it was also observed in 8% of patients in the remission group. This suggests the need to understand the influence of subsequent treatment options on the remission (Table 1). Therefore, treatment choices for UC are adjusted based on changes in the disease status. Thus, the findings observed during enrollment may not necessarily predict remission at the final 3-year follow-up. In this study, we developed a predictive model using machine learning to analyze the pathological changes and treatment decisions made from registration up to 2 years later, to predict remission at the 3-year mark by changes in the disease state; therefore, findings at registration do not necessarily translate into remission at the final 3-year follow-up.

In this study, we constructed a model that can predict remission at 3 years by machine learning pathological transitions and treatment choices from registration to 2 years later.

Traditional statistical methods rely primarily on linear or logistic regression to analyze the relationship between various variables and outcomes. By contrast, machine learning techniques can utilize neural networks to derive a wider array of standard variables [27]. However, conventional machine learning methods are often limited in their ability to visualize these processes [28]. Therefore, we used a machine learning-based method based on a PWL model [15,19,29], which allows for correlations between each explanatory variable and the target variable. Our model divides initial CS users into groups with high and low remission rates by estimating the probability of remission induction at 3 years. Furthermore, in the PWL model, positive weight values for each variable positively correlated with remission, whereas negative values negatively correlated with remission. The absolute weight value indicated the strength of the prediction [15,19].

In this study, we developed three distinct prediction models: the first, a model relying on data from the year of registration (model_0y); the second, a model utilizing data from the registration year extending into the following year (model_1y), and the third, a model based on data from the registration year up to 2 years later (model_2y). We evaluated the accuracy of each model, and as illustrated in Figure 3, the AUC increased from 0.628 to 0.774 with the addition of explanatory variables.

Nevertheless, whether increasing the number of explanatory variables would lead to overfitting remained a valid concern. However, throughout all tests, no significant decrease was observed in test precision compared with training precision. This suggests that the risk of overlearning was effectively mitigated. Figure 4 shows a comparison of the three prediction models. In each model, the presence of pseudopolyps at registration demonstrated the highest correlation with remission in the analysis of significant factors (scores). Notably, a negative median value of the regression coefficient (weight) was associated with each important factor. This indicates that the absence of pseudopolyps is the most critical factor for achieving remission after 3 years. Concerning treatment selection, the following factors were identified as important for remission at the 3-year mark: 5-ASA administration at the time of registration in model_0y (Figure 4(A)), surgery at the time of registration, CAP implementation in model_1y (Figure 4(B)), and the absence of CS administration in the year following registration in model_2y (Figure 4(C)).

The prediction made using model_2y was regarded as the most dependable, as it exhibited the highest AUC. In this model, the administration of CS after 1 year of enrollment was found to be negatively associated with remission. This finding aligns with the trend observed in Table 2, which indicates that higher remission rates were present in treatment categories with lower CS administration following 1 year of enrollment.

Pseudopolyps are characteristic features of the intestinal wall linked to chronic inflammation and are reported to be present in 10%–40% of UC cases [30]. Additionally, individuals with pseudopolyps are at heightened risk of requiring treatment escalation, including the use of biological agents or surgical interventions [31]. In this context, the significance of pseudopolyps as an influential factor affecting remission in the predictive model is noteworthy.

When examining the duration from onset to registration based on the presence of pseudopolyps, the average time was 4.0 ± 7.7 years (95% CI: 3.3–4.7 years) for those with pseudopolyps, compared to 2.0 ± 4.7 years (95% CI: 1.8–2.2 years) for those without. There was a statistically significant tendency for the time from onset to registration to be longer in cases involving pseudopolyps (*p* < 0.001), suggesting that the presence of pseudopolyps at the time of registration reflects inadequate treatment outcomes.

The limitations of this study include its reliance on the UC medical information collected annually by the government. The available data were restricted to the age of onset, age at registration, findings on barium, endoscopy, biopsy examinations, and several blood tests, including red blood cell (RBC), hemoglobin (Hb), WBC, ESR, CRP, TP, and Alb (Supplementary Table S1). Consequently, data on the bilirubin levels, blood coagulation function, and stool calprotectin levels could not be obtained. Additionally, the information on treatment was limited to drug and surgical interventions, and nutritional therapy data could not be determined. This study was limited to the period from 2003 to 2014. Since anti-tumor necrosis factor alpha (TNFα) antibody treatments for ulcerative colitis became available in Japan in 2010, the effects of these antibodies are not represented in the newly recorded cases from 2003 to 2006.

## 5. Conclusions

In this study, we developed a machine learning model to predict remission 3 years after initiating remission induction therapy in patients with UC who required CS administration. The accuracy of the model was enhanced without the risk of overfitting by incorporating clinical data collected up to 2 years after registration. Furthermore, all predictive models indicated that the presence of pseudopolyps at the time of registration inhibited successful remission induction. In conditions characterized by cycles of inflammation and remission such as UC, the influence of time-series data must be considered when forecasting long-term outcomes. However, traditional statistical methods have certain limitations. In this context, the PWL model is advantageous for predicting the prognosis and identifying the factors that influence it.

## Supplementary Material

Supp materials_revised.docx

## Data Availability

All the patients provided written informed consent to participate in the Specified Disease Treatment Research Program of the MHLW. After collection of informed consent forms and approval by a review committee in the respective prefectural governments, personal information was anonymized, and cases were registered in the MHLW database. The MHLW provided anonymized data for analysis (March 9, 2021). Data supporting the findings of this study are available from the corresponding author upon reasonable request.
