# Supplementary material for: Explainable artificial intelligence for prediction of refractory ulcerative colitis: analysis of a Japanese Nationwide Registry
Source: Ann Med. 2025 May 5;57(1):2499960. doi: 10.1080/07853890.2025.2499960 (PMC12054586; doi:10.1080/07853890.2025.2499960)
Supplement: Supp materials_revised.docx [file IANN_A_2499960_SM4782.docx]

**Explainable artificial intelligence for prediction of refractory ulcerative colitis: Analysis of**

**a Japanese nationwide registry**

Masaya Sano^a^, Yasuhiro Kanatani,^b^ Takashi Ueda,^a^ Shota Nemoto,^c^ Yurin Miyake,^b^ Naoko Tomita,^b^ Hidekazu Suzuki,^a^

**Affiliations:**

1. Department of Gastroenterology, Tokai University School of Medicine, 143 Shimokasuya, Isehara, Kanagawa, Japan
2. Department of Clinical Pharmacology, Tokai University School of Medicine, 143 Shimokasuya, Isehara, Kanagawa, Japan
3. Industrial & Digital Business Unit, Hitachi, Ltd., 1-5-2 Sotokanda, Chiyoda-ku, Tokyo, Japan

**Diagnostic criteria for ulcerative colitis (UC)**

A diagnosis is confirmed when the following conditions are met: (1) is present, one of the items in (2) is satisfied, (3) is present, and the diseases in (4) can be ruled out.

(1) Clinical symptoms

Persistent or recurrent mucous/bloody stool or a history of such stool.

(2) Imaging findings

1) Endoscopy

(a) Diffuse involvement of the mucosa, with a coarse or fine granular appearance, brittleness, and easy bleeding, along with mucous and purulent secretions or

(b) Multiple erosions, ulcers, or pseudopolyps

2) Intestinal radiography

(a) Diffuse changes on the mucosal surface, such as plasticity or fine granules, or

(b) Multiple erosions, ulcers, or pseudopolyps, along with disappearance of the haustrum

and narrowing or shortening of the intestinal canal.

(3) Biopsy histology

Diffuse inflammatory cell infiltration, mainly in the inner layer of the mucosa, with decreased or absent goblet cells, erosion, crypt abscess, and abnormal glandular arrangement. Even if the examination of (2) and (3) is insufficient or cannot be performed, if gross and histological findings and characteristics of ulcerative colitis are seen during resection surgery or autopsy, the diagnosis is considered to be defined if other diseases (4) can be ruled out.

(4) Diseases that should be ruled out include infection enteritis, such as bacterial dysentery, amoebic dysentery, schistosomiasis japonica, tuberculosis coli, Campylobacter enterocolitis, radiation colitis, ischemic colitis, drug-induced colitis, Crohn’s disease, Bechet’s intestinal type, and lymph-follicular-proliferative diseases.

Note:

- In some rare cases, patients may not be aware of hemoptysis, so careful consideration is needed.

- If findings are mild and the diagnosis is not specific, it should be treated as a 'suspicious diagnosis' until definite findings are obtained.

**Table S1. The application form of UC**

| Item |  |  | | |  | |  | Option |
| --- | --- | --- | --- | --- | --- | --- | --- | --- |
| Application year |  |  | | |  | |  | YYYY(AD) |
| Gender |  |  | | |  | |  | 1:Man 2:Woman |
| Birthday | Year |  | | |  | |  | YYYY(AD) |
|  | Age |  | | |  | |  |  |
| Onset date | Year |  | | |  | |  | YYYY(AD) |
|  | Age |  | | |  | |  |  |
| Date of first visit to a doctor | |  | | |  | |  | YYYYMMDD（AD） |
| Family history |  |  | | |  | |  | 1: Yes 2: No 3: Unknown |
| Pathological condition | Clinical course | | | | 1. First attack | | | 1: Yes |
|  |  |  | | | 2. Relapse and remission | | | 1: Yes |
|  |  |  | | | 3. Chronic persistent | | | 1: Yes |
|  |  |  | | | If 1 is selected. | | | 1: First 2: Relapse |
|  |  |  | | | 4. Acute fulminant | | | 1: Yes |
|  |  |  | | | 5. Unknown | | | 1: Yes |
|  | No. of hospitalizations | | | | Total | |  | Current facility + other facilities |
|  |  |  |  |  | Current facility | | |  |
|  |  |  |  |  | Other facilities | | |  |
|  | Refractory |  | | |  | |  | 1: Yes 0: No |
|  |  |  | | | 1. Active for more than  6 months | | | 1: Yes |
|  |  |  | | | 2: Relapse at least twice  a year | | | 1: Yes |
| Severity | Date of evaluation | | | |  | |  | YYYYMMDD（AD） |
|  | Severity |  | | |  | |  | 1: mild 2: moderate 3: severe 4: fulminant  5: unknown |
| Extent of the lesion | Latest affected area | | | | Date of evaluation | | | YYYYMMDD（AD） |
|  |  |  | | | 1. Rectum | | | 1: Yes |
|  |  |  | | | 2. Colon | |  | 1: Yes |
|  |  |  | | |  | 1. Sigmoid | | 1: Yes |
|  |  |  | | |  | 2. Descending | | 1: Yes |
|  |  |  | | |  | 3. Transvers | | 1: Yes |
|  |  |  | | |  | 4. Ascending | | 1: Yes |
|  |  |  | | | 3. Cecum | | | 1: Yes |
|  |  |  | | | 4. Ileum | |  | 1: Yes |
|  |  |  | | | 5.Unknown | | | 1: Yes |
|  | Intestinal complications | | | | | |  | 1: Yes 2: No |
|  |  |  | | | If 1 is selected. | | | Describe the illness. |
|  | Family occurrence of UC | | | | | |  | 1: Yes 2: No |
|  |  |  | | | If 1 is selected. | | | Describe the relationship. |
|  | Family occurrence of Crohn disease | | | | | | | 1: Yes 2: No |
|  |  |  | | | If 1 is selected. | | | Describe the relationship. |
| Internal treatment | Current therapy | | | | 1. Steroids | | | 1: Yes 2: No |
|  | (Including those associated with postoperative complications.) | | | | 2. Intravenous hyperalimentation | | | 1: Yes 2: No |
|  |  |  |  |  | 3. 5-ASA | | | 1: Yes 2: No |
|  |  |  |  |  | 4. Immunosuppressants | | | 1: Yes 2: No |
|  |  |  |  |  | 5. Other | |  | 1: Yes 2: No |
|  |  |  |  |  | If 1 is selected. | | | Describe the name of drugs. |
|  | Adverse events | | | |  | |  | 1: Yes 2: No |
|  |  |  | | | If 1 is selected. | | | Describe symptoms and findings |
| Surgical treatment | Reason for surgery | | | | 1. Bleeding | | | 1: Yes |
|  |  |  | | | 2. Megacolon | | | 1: Yes |
|  |  |  | | | 3. Cancer | | | 1: Yes |
|  |  |  | | | 4. Perforation | | | 1: Yes |
|  |  |  | | | 5. Resistant for therapy | | | 1: Yes |
|  |  |  | | | 6. Extraintestinal complications | | | 1: Yes |
|  |  |  | | | If 1 is selected. | | | Describe the detail. |
|  |  |  | | | 7. Other | |  | 1: Yes |
|  |  |  | | | If 1 is selected. | | | Describe the detail. |
|  |  |  | | |  | | |  |
|  | Date / Surgical method | | | | 1st | | date of surgery | YYYYMMDD（AD） |
|  |  |  | | |  | | Surgical method | Describe the detail. |
|  |  |  | | | 2nd | | date of surgery | YYYYMMDD（AD） |
|  |  |  | | |  | | Surgical method | Describe the detail. |
|  | Postoperative complication | | | | | |  | 1: Yes 2: No |
|  |  |  | | | If 1 is selected. | | | Describe the detail. |
|  | Macroscopic and histological findings | | | | | | | Describe the detail. |
| Fecal pathogen microorganism examination | Date |  | | |  | |  | YYYYMMDD（AD） |
|  |  |  | | |  | |  | 1: Yes 2: No |
|  | Name of microorganism | | | | | If 1 is selected. | | Describe the detail. |
| Clinical symptoms and findings | Recent findings | Hight (cm) | | | | |  |  |
|  |  | Weight (Kg) | | | | |  |  |
|  |  | 1. Main symptoms | | | | | | |
|  |  |  | Date | | | |  | YYYYMMDD（AD） |
|  |  |  | | (1) Stool frequency (times/day) | | | |  |
|  |  |  | | (2) Status of stool | | | |  |
|  |  |  | |  | | | Bleeding | 1: No 2: slightly 3: moderate 4: severe |
|  |  |  | |  | | | Appearance | 1: solid 2: muddy 3: watery |
|  |  |  | | (3) Abdominal symptoms | | | |  |
|  |  |  | |  | | | Spontaneous pain | 1: Yes 2: No |
|  |  |  | |  | | | Location | Describe the detail. |
|  |  |  | | (4) Body temperature (℃） | | | |  |
|  |  |  | | (5) Pulse rate (/min) | | | |  |
|  |  | 2. Blood test | | | | |  |  |
|  |  |  | | Date | | |  | YYYYMMDD（AD） |
|  |  |  | | Implementation of examination | | | | 1: Yes 2: No |
|  |  |  | | (1) RBC （×10^4^ /mm^3^） | | | |  |
|  |  |  | | (2) Hemoglobin (g/dl) | | | |  |
|  |  |  | | (3) WBC (/mm^3^) | | | |  |
|  |  |  | | (4) ESR (mm) | | | |  |
|  |  |  | | (5)CRP (mg/dl) | | | |  |
|  |  |  | | (6) Total protein (g/dl) | | | |  |
|  |  |  | | (7) Albumin (g/dl) | | | |  |
|  |  | 3. Colonoscopically findings | | | | | |  |
|  |  |  | | Date | | |  | YYYYMMDD（AD） |
|  |  |  | | Implementation of examination | | | | 1: Yes 2: No |
|  |  |  | | (1) Continuous lesion | | | | 1: Yes 2: No |
|  |  |  | | (2) Disappearance of haustra | | | | 1: Yes 2: No |
|  |  |  | | (3) Mucosal fragility | | | | 1: Yes 2: No |
|  |  |  | | (4) Erosion/ulcer | | | | 1: Yes 2: No |
|  |  |  | | (5) Pseudopolyps | | | | 1: Yes 2: No |
|  |  | 4. Endoscopically findings | | | | | |  |
|  |  |  | | Date | | |  | YYYYMMDD（AD） |
|  |  |  | | Implementation of examination | | | | 1: Yes 2: No |
|  |  |  | | (1) Loss of vascular markings | | | | 1: Yes 2: No |
|  |  |  | | (2) Easy bleeding | | | | 1: Yes 2: No |
|  |  |  | | (3) Mucosal fragility | | | | 1: Yes 2: No |
|  |  |  | | (4) Erosion/ulcer | | | | 1: Yes 2: No |
|  |  |  | | (5) Pseudopolyps | | | | 1: Yes 2: No |
|  |  |  | | (6) Continuous lesion | | | | 1: Yes 2: No |
|  |  | 5. Biopsy findings (pathological examination) | | | | | | |
|  |  |  | | Date | | |  | YYYYMMDD（AD） |
|  |  |  | | Implementation of examination | | | | 1: Yes 2: No |
|  |  |  | | (1) Cell infiltration | | | | 1: Yes 2: No |
|  |  |  | | (2) Erosion | | | | 1: Yes 2: No |
|  |  |  | | (3) Cryptal abscess | | | | 1: Yes 2: No |
|  |  |  | | (4) Reduced no. of goblet cells | | | | 1: Yes 2: No |
|  |  |  | | (5) Mucosal abnormality | | | | 1: Yes 2: No |
|  |  |  | | (6) Dysplasia | | | | 1: Yes 2: No |
| Differential diagnosis |  | (1) Infectious colitis | | | | | | 1: Can be distinguished  2: Cannot be distinguished |
|  |  | (bacterial dysentery, amoebic dysentery, schistosomiasis japonica, tuberculosis of the colon, campylobacter enteritis, etc.) | | | | | |  |
|  |  |  |  |  |  |  |  |  |
|  |  |  |  |  |  |  |  |  |
|  |  |  |  |  |  |  |  |  |
|  |  | (2) Radiational colitis | | | | | | 1: Can be distinguished  2: Cannot be distinguished |
|  |  | (3) Ischemic colitis | | | | | | 1: Can be distinguished  2: Cannot be distinguished |
|  |  | (4) Drug-induced colitis | | | | | | 1: Can be distinguished  2: Cannot be distinguished |
|  |  | (5) Crohn disease | | | | | | 1: Can be distinguished  2: Cannot be distinguished |
|  |  | (6) Colon Bechet | | | | | | 1: Can be distinguished  2: Cannot be distinguished |
|  |  | (7) Lymph proliferative disorders | | | | | | 1: Can be distinguished  2: Cannot be distinguished |

**Table S2. Datasets used in the pointwise linear model.**

|  |  | Dataset | | |  |  |  | Dataset | | |
| --- | --- | --- | --- | --- | --- | --- | --- | --- | --- | --- |
| Feature | Type | 0y | 1y | 2y |  | Feature | Type | 0y | 1y | 2y |
| Gender | B | × |  |  |  | Ba. continuous lesion | B | × |  |  |
| Age | Q | × |  |  |  | Ba. loss of haustra | B | × |  |  |
| Age onset | Q | × |  |  |  | Ba. rough mucosa | B | × |  |  |
| Daily life | Q | × | × | × |  | Ba. erosion/ulcer | B | × |  |  |
| Severity stage | Q | × | × | × |  | Ba. pseudo-polyps | B | × |  |  |
| Lesion rectum | Q | × |  |  |  | Ed. loss of vascular markings | B | × | × | × |
| Lesion colon | B | × |  |  |  | Ed. easy bleeding | B | × | × | × |
| Lesion sigmoid colon | B | × |  |  |  | Ed. mucosal friability | B | × | × | × |
| Lesion descending colon | B | × |  |  |  | Ed. erosion | B | × | × | × |
| Lesion transverse colon | B | × |  |  |  | Ed. pseudo-polyps | B | × | × | × |
| Lesion ascending colon | B | × |  |  |  | Ed. continuous lesion | B | × | × | × |
| Lesion cecum | B | × |  |  |  | Bp. cell infiltration | B | × | × | × |
| Lesion ilium | B | × |  |  |  | Bp. erosion | B | × | × | × |
| Lesion unknown | B | × |  |  |  | Bp. cryptal abscess | B | × | × | × |
| Distribution (E1,E2,E3,other) | C | × |  |  |  | Bp: reduced no of goblet cells | B | × | × | × |
| Stool bacteria | B | × |  |  |  | Bp. mucosal abnormalities | B | × | × | × |
| Height | Q | × |  |  |  | Bp. dysplasia | B | × | × | × |
| Body weight | Q | × |  |  |  | Tx. corticosteroids | B | × | × | × |
| Stool frequency | Q | × | × | × |  | Tx. intravenous hyperalimentation | B | × | × | × |
| Stool bleeding | Q | × | × | × |  | Tx. 5-ASA | B | × | × | × |
| Stool status | Q | × |  |  |  | Tx. immunosuppressant | B | × | × | × |
| Spontaneous abdominal pain | B | × |  |  |  | Tx. CAP | B | × | × | × |
| Body temperature | Q | × | × | × |  | Tx. anti-TNFα antibodies | B | × | × | × |
| Pulse | Q | × | × | × |  | Surgery for bleeding | B | × | × | × |
| RBC | Q | × |  |  |  | Surgery for cancer | B | × | × | × |
| Hb | Q | × | × | × |  | Surgery for megacolon | B | × | × | × |
| WBC | Q | × |  |  |  | Surgery for perforation | B | × | × | × |
| ESR | Q | × | × | × |  | Surgery for resistance | B | × | × | × |
| CRP | Q | × | × | × |  | Mayo Score | Q | × | × | × |
| TP | Q | × |  |  |  |  |  |  |  |  |
| Alb | Q | × |  |  |  |  |  |  |  |  |

Note: binary variable; B, quantitative variable; Q, category variable; C, barium enema examination; Ba, endoscopic examination; Ed, biopsy examination; Bp, therapy; Tx.

**Table S3. Best hyperparameters of the pointwise linear model for clustering of UC.**

|  | model_0y | model_1y | model_2y |
| --- | --- | --- | --- |
| Number of epochs | 190 | 200 | 210 |
| Number of inner layers | 5 | 5 | 5 |
| Size of layers | 150 | 240 | 170 |
| Level smoothing | 0.19×10^-2^ | 0.10 | 2.45×10^-2^ |
| Learning rate | 1.0×10^-3^ | 3.33×10^-5^ | 7.81×10^-5^ |
| Momentum | 0.956 | 0.960 | 0.964 |
| Optimization | Adam | Adam | Adam |
| Dropout rate of inner layers | 0.489 | 0.140 | 0.080 |
| Dropout rate of input layers | 0.496 | 0.044 | 0.237 |
| Regularization coefficient | 1.93×10^-10^ | 3.41×10^-7^ | 3.21×10^-7^ |
